# Experimental Investigation into Bipolar Sealability of Elastin-Rich Tissue

**DOI:** 10.3390/medsci14030378

**Published:** 2026-07-07

**Authors:** Andreas Kirschbaum, Gesche Schmidt, Moritz Jesinghaus, Nikolas Mirow

**Affiliations:** 1 Department of Visceral, Thoracic, and Vascular Surgery, University Hospital Giessen and Marburg (UKGM), Marburg Site, D-35047 Marburg, Germany; 2 Department Human Medicine, Philipps University of Marburg, D-35047 Marburg, Germany; geschemarleen@gmail.com (G.S.);; 3 Institute of Pathology, University Hospital Giessen and Marburg (UKGM), Marburg Site, D-35047 Marburg, Germany; moritz.jesinghaus@uk-gm.de

**Keywords:** bipolar vessel sealing, elastin-rich tissue, porcine aortic model, tissue compressibility, peel force testing, elastin thermal stability, impedance-controlled sealing

## Abstract

Background: Elastin-rich vessels such as the aorta pose a particular challenge for bipolar tissue sealing due to their high elasticity and low compressibility. It is unclear under which conditions a mechanically resilient seal can be achieved in such tissues. The aim of this experimental study was to investigate the sealability of elastin-rich aortic wall strips from pigs. The influence of compression pressure and impedance control on the resulting peel forces is analyzed. Methods: Porcine aortas were harvested immediately postmortem, segmented, and histologically characterized (hematoxylin–eosin (HE) and elastin–Van Gieson (EVG). Mechanical compressibility was determined uniaxially on full-wall disks (Ø 10 mm) and compared with carotid arteries Ø < 5 mm. Aortic strips (5 × 25 mm) were sealed bipolarly, first with a commercially available instrument. Further seals were performed employing a specially developed device that allowed defined compression pressures (2.5–10 kg) and variable final impedance settings (250 vs. 500 Ω). Sealing quality was quantified via peel force measurements. Statistical analysis was performed by Mann–Whitney U tests. Results: The aortic wall showed a high elastin content (42.8 ± 3.4%) and low compressibility (only 44.9% at 490 N), significantly lower than in carotid arteries (92.8% at 490 N). The standard instrument produced no stable seals at all. However, defined compression in the special setup led to significant improvements: mean peel force increased from 0.09 N (2.5 kg) to 0.71 N (10 kg; +68.8%). Increasing the interval between the initial and final impedance setting (500 Ω) further increased the peel force by 25.5% (0.32 ± 0.05 N) at 2.5 kg compression. Combining compression of 5 kg and 500 Ω final impedance interval achieved the highest stability (0.91 ± 0.2 N; *p* < 0.001). Histologically, vessel wall structures were not destroyed by the process; they remained largely intact. Coagulation zones were limited to the contact areas. Conclusions: Resilient bipolar sealing of elastin-rich aortic wall tissue is fundamentally possible but requires defined mechanical compression and adapted impedance control. As this study relied exclusively on ex vivo peel-force measurements, the findings should be interpreted as mechanistic rather than clinically validated. Functional assessments, such as burst pressure testing and evaluation in intact vessel segments, will be required in future work.

## 1. Introduction

Bipolar tissue and vessel sealing has become a key technology in modern surgery over the past decades [1,2,3,4,5]. It enables fast, reliable, and reproducible hemostasis, reduces the need for ligatures, and contributes significantly to the efficiency of minimally invasive procedures [4,6,7,8]. The technology works by combining mechanical compression with controlled energy application, leading to thermally induced denaturation and reconfiguration of structural proteins. Collagen, which loses its triple helix structure at temperatures between 60 and 90 °C and then solidifies in a reorganized form, forms the basis for stable sealing sutures [9,10,11]. The effectiveness of this technology has been extensively documented for collagen-rich, highly compressible tissues such as smaller arteries, veins, or parenchymal organs [12,13]. In contrast, elastin-rich tissues, especially large arteries such as the aorta, are the focus of increasing scientific attention [14,15,16,17]. Elastin, unlike collagen, is a highly structured, extremely elastic protein, which has significantly higher thermal stability and undergoes structural changes at temperatures above 150 °C [18]. These properties make it difficult to form a homogeneous coagulation zone and limit the mechanical strength of a sealing seam. Furthermore, vessels rich in elastin exhibit pronounced resilience and low compressibility, which makes it difficult to achieve the necessary compression of tissue layers between electrodes [15,19,20,21]. Despite clinical relevance, only a few systematic studies concerning sealability of elastin-rich vessels have been published to date [22,23]. They mostly focus on collagen-rich structures or vessels with thin walls, where the mechanical and thermal conditions for successful sealing are relatively favorable. Not all vessels, of course, exhibit favorable properties in this regard. One characteristic representative of this is the aorta. Sealing options of this central vessel are nevertheless of interest. The aorta has a pronounced wall thickness and little collagen, but high elastin content. The media is rich in elastic lamellae, which are responsible for the typical windkessel function of large arteries. These elastic structures significantly determine the mechanical properties of the aorta and influence both its compressibility and its thermal response to energy input. Understanding these prerequisites for successful elastin-rich tissue sealing is a key step. Sufficient compression is necessary to bring the tissue layers into close contact, improve electrical conductivity, and form a homogeneous coagulation zone. At the same time, compression must not be overly forceful to avoid structural damage or uncontrolled lateral displacement of tissue layers.

An important factor is impedance control of modern sealing systems. Impedance of tissue changes during energy application depending on temperature, water content, and protein structure. Many available devices use these changes to automatically regulate energy flow and determine the time at which sealing is complete. In the case of elastin-rich tissues, however, it is questionable whether standardized impedance thresholds are sufficient to create a stable coagulation zone or whether parameters need to be adjusted. Initial evidence from the literature [24,25,26,27] suggests that greater intervals between the initial and final resistances of the tissue may lead to more intense thermal denaturation, making a difference in elastin-rich structures.

Against this background, the present study pursues two central objectives: First, it aims to investigate the extent to which the compressibility of an elastin- rich tissue, such as the aortic wall, differs from that of more collagen-rich vessels, such as the carotid artery. Second, it aims to systematically analyze whether and under what conditions resilient bipolar sealing of elastin-rich aortic wall strips is at all possible. The influences of different compression pressures and of varying differences between initial and final resistance on resulting peel forces are investigated. In addition, histological analyses are performed to characterize structural changes in the vessel walls and sealing zones. Mechanical results could thus become histologically substantiated.

For practical reasons, an ex vivo model using pig aortas was chosen. This material [28,29,30,31] is well-suited for experimental studies due to the structural similarity to human aortas.

It was not intended to transfer the data obtained from this study directly into clinical practice. This final goal will require several further steps. Instead, the work presented aimed at an improved understanding of biomechanical and thermal principles in tissue-sealing. This present work focuses on isolated tissue strips and peel-force testing. It represents an exploratory mechanistic study rather than a functional validation of vascular sealing performance.

## 2. Materials and Methods

In freshly slaughtered pigs (EU standard: 90 kg), the aorta and the carotids were separated after removal of the entire heart–lung blocks. Preparations were rinsed and transported to our laboratory and cooled in a moist compress at 4 °C. Transport time was a maximum of 10 min. In the laboratory, the aortas were divided into 2.5 cm-long sections. The thickness of the 8 aortic and carotid walls was determined using a digital gauge. In addition, samples from the aortic walls were sent to the Institute of Pathology for histological examination. Hematoxylin–eosin (HE) and elastin–Van Gieson (EVG) staining of the aortic walls were performed. HE and EvG staining of 8 carotid arteries were performed to allow for direct comparison between the elastin-rich aorta and the collagen-rich carotid artery. The proportion of elastin and collagen was determined from the digitized histology images by color analysis of the characteristic staining (GSA Image Analyzer Version 4.4.1, www.gsa-online.de (URL accessed on 3 October 2026)). Histological assessment was intentionally qualitative and aimed at identifying structural preservation and localization of coagulation zones. Quantitative morphometric analyses (e.g., coagulation depth, elastin fragmentation, collagen remodeling) were beyond the scope of this study but are planned for future work.

### 2.1. Uniaxial Compression Tests

**Figure 1:**
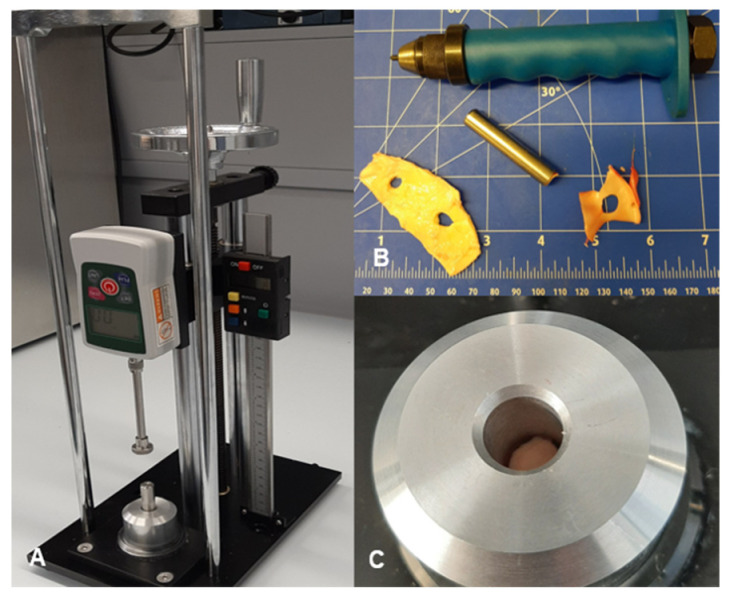
Experimental setup for investigating compression (**A**). Device for investigating the compressibility of a tissue disk (**B**). View of punched-out tissue disks from aortic walls (**C**). Visible channel in the metal cylinder with an inserted tissue disk (own images).

The uniaxially limited compression was examined in all 8 aortic and carotid wall preparations. Full-wall disks were cut out of the aortic and carotid walls using a punch (diameter: 10 mm). For all compression experiments, the intima and adventitia of specimens were carefully kept intact. None were mechanically removed to preserve the full aortic thickness. The disks were placed in a cylinder with a central channel of 10 mm in diameter. A pin was inserted into this channel, and the disk was gradually compressed with increasing force. The decreasing height of the disk was indicated by a digital measuring device. Figure 1 illustrates the experimental setup. For evaluation, the average percentage compression was compared to the compression force. Results were compared to the carotid arteries.

### 2.2. Bipolar Sealing of Aortic Strips Using a Commercially Available Sealing Instrument

**Figure 2:**
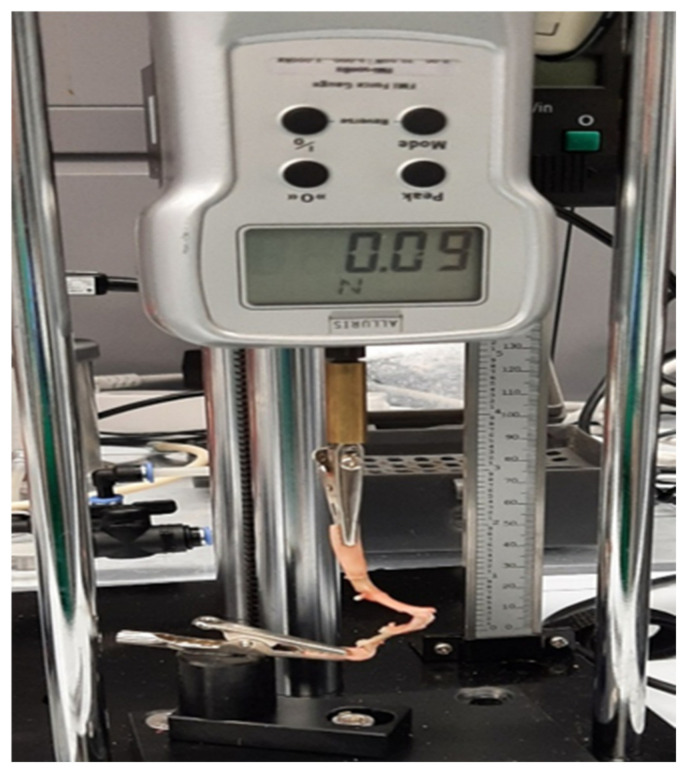
A sealing seam was pulled vertically upwards apart. Structural failure occurred exclusively within the sealing zone; there were no cracks or slippage around the clamps. The digital display remains at maximum peel pressure (own photo).

Straight strips measuring 5 × 25 mm were cut from the aortic walls. These strips were laid on top of each other and fixed at both ends to a corkboard using pins. First bipolar sealing was initiated with a commercially available bipolar sealing device, marSeal^®^ 5 plus (KLS Martin SE & Co. KG, Tuttlingen, Germany), connected to the maXium^®^ sealer generator (KLS Martin SE & Co. KG, Tuttlingen, Germany). Eight sealing processes were executed in this manner. Sealed strips were clamped into a specific device to determine peel force. Peel force was defined as the force at which the strips can be separated by vertical traction. In our setup, this force was displayed digitally. Figure 2 shows an overview of the basic test setup for determining peel forces.

### 2.3. Bipolar Sealing of Aortic Strips with a Proprietary Sealing Structure

**Figure 3:**
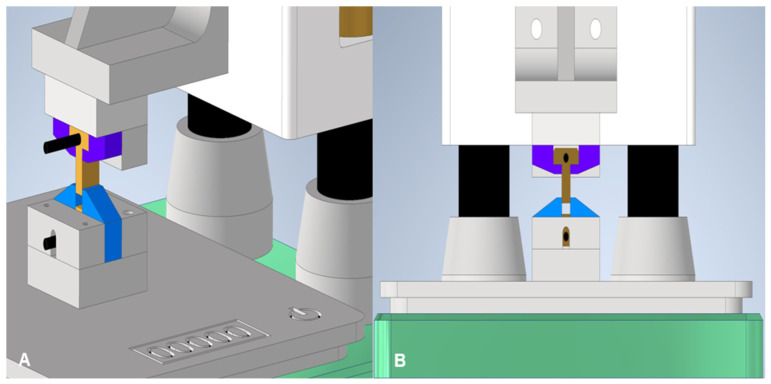
Experimental setup designed using virtual construction software for sealing tissue strips (**A**): view on the construction from the side (**B**): front view of the construction (design by Precision Engineering Workshop Fb 20 Philipps University of Marburg).

An individual apparatus was designed and built to investigate factors influencing the quality of the sealing process. A special feature is a channel inside which lateral tissue movement caused by pressure was reduced. This compression channel was 5 mm in width and 5 mm in depth. The casing was coated with non-conductive material to minimize lateral thermal expansion. Electrodes were solid brass. Figure 3 shows the configuration of the electrodes, made from solid brass. This created a uniform contact zone between the electrodes and the aortic wall strips. With this setup, we performed seals at different compression levels, which were continuously displayed on a scale. Before each sealing, the digital scale was calibrated to 0.

**Figure 4:**
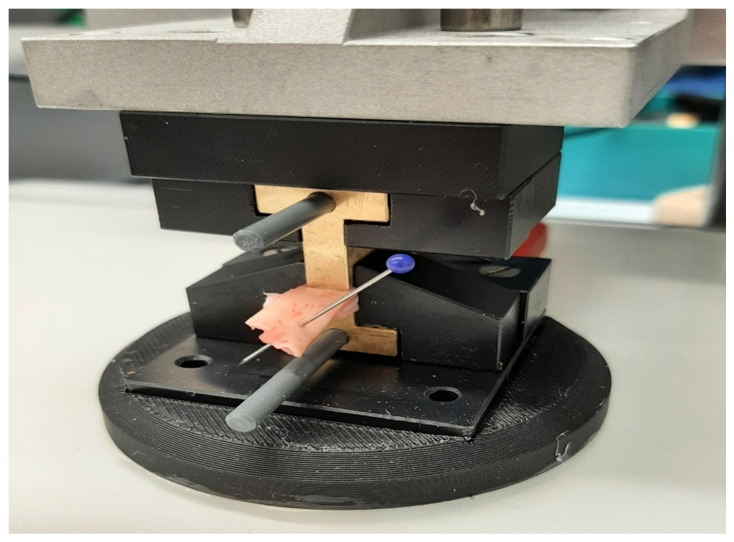
Experimental setup. Inserted aortic wall strips are compressed in the shaft by electrodes. (Own photograph).

Figure 4 shows an overview of the experimental setup. Twelve superimposed aortic strips were examined per group. Compression pressures started at 2.5 kg, rising to 5 kg, 7.5 kg, and up to 10 kg. In addition, tests were carried out in which the sealing procedure was not terminated at the clinically often employed elevation of impedance by 250 Ω, but at levels of 500 Ω. Specimens were sent to the Institute of Pathology for histological examination of the sealing sutures (HE and EVG staining). After each sealing, the peel force was measured as described above. The mean values of the individual groups were compared using a nonparametric Mann–Whitney U test. A significant difference was found at *p* < 0.05. Data analysis was performed using the numiqo program (www.numiqo.de) (URL accessed on 5 March 2026).

## 3. Results

**Figure 5:**
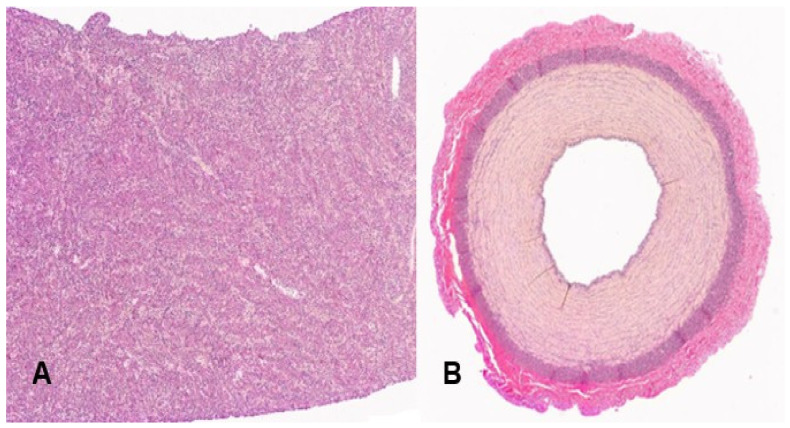
(**A**): Aortic wall in EVG staining (4× magnification). (**B**): Cross-section of the porcine carotid in EVG Staining (4× magnification) (Institute of Pathology, Marburg).

The mean thickness of the aortic walls (*n* = 8) was 1.84 ± 0.5 mm, and the mean proportion of elastin in the histological section was 42.83 ± 3.4%. The mean proportion of collagen analyzed was 22.6 ± 2.1%. The mean thickness of the carotid wall (outer diameter: 5.2 ± 0.4 mm) was 0.59 ± 0.01 mm, the mean proportion of elastin in the histological section was 7.3 ± 2.1%, and the mean proportion of collagen analyzed was 20.5 ± 1.8%. Figure 5 shows a typical histological image of the aortic and carotid wall with purple-stained elastin fibers in EVG staining.

**Figure 6:**
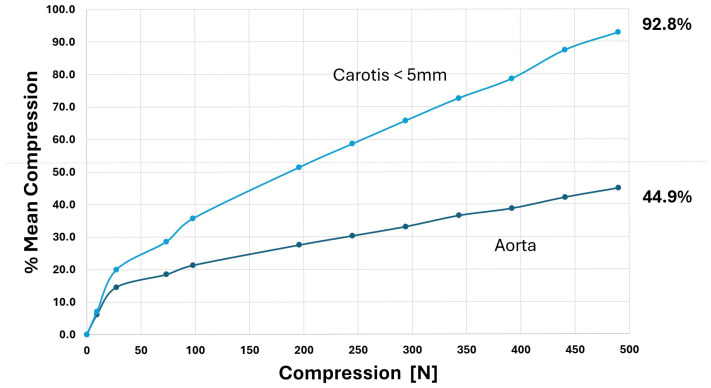
Overview of the mean % compression of the aortic walls (*n* = 12) compared to carotid arteries with an outer diameter of less than 5 mm (*n* = 12) (own results).

At a compression force of 490 N, the aortic wall disks were compressed by 44.9%, compared to the carotid arteries, which were compressed by 92.8% at the same force. Table 1 shows an overview of measurements. The difference is particularly evident in Figure 6, where all measured values are plotted in a graph.

**Figure 7:**
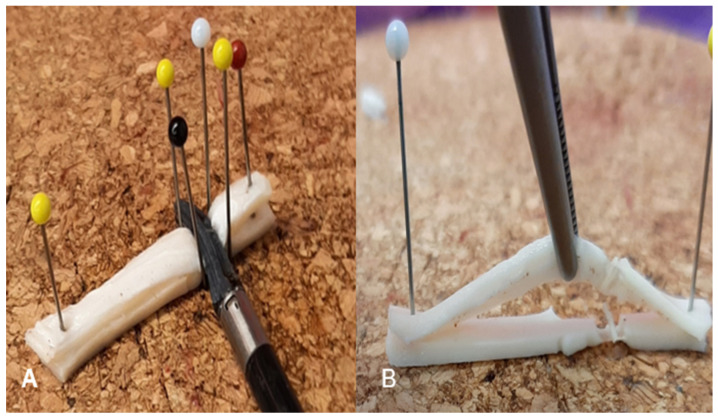
Bipolar sealing with the marSeal^®^ 5 plus sealing instrument of two superimposed aortic wall strips (**A**). Start of sealing with the sealing instrument closed. To prevent displacement of the aortic wall strips after closing the device, the walls were fixed with additional pins. (**B**): Result of the sealing: the upper strip can be lifted very easily with forceps and has come loose (own image).

Eight doubled aortic wall strips were sealed with a conventional bipolar sealing instrument connected to a generator. At inspection, none of the sealings were successful. Lifting any sealed strip without force and only very slightly with tweezers led to immediate rupture. Figure 7 illustrates this situation. It was therefore quite impossible to measure peel forces. This complete failure to obtain stable tissue seals highlights the limitations of current bipolar technology if used on thick-walled, elastin-rich vessels.

**Figure 8:**
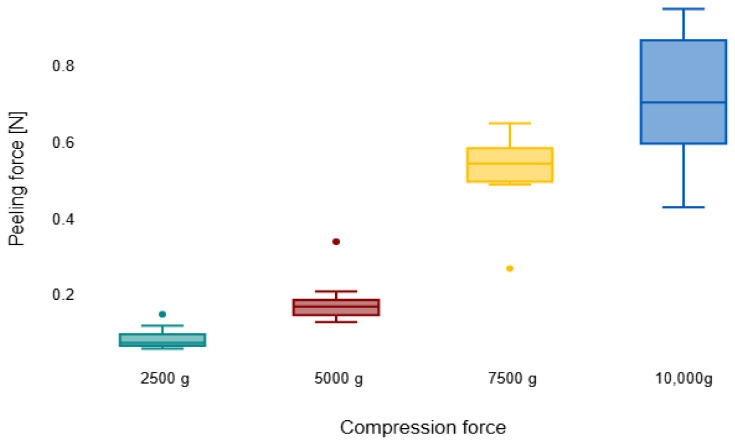
Box plot of the peeling forces [N] of the individual groups (*n* = 12 in each case) as a function of the compression forces (own results).

Experiments were repeated using the specific device we had constructed. The aortic wall strips fixed with pins were placed in the slot provided, and a mobile electrode was lowered until the intended compression pressure was reached. Average peel forces were determined. Increasing the compression force from 2.5 kg to 10 kg led to average peeling forces from 0.09 N up to 0.71 N, an increase of +68.8%. Table 2 and Figure 8 show an overview of the results. In all tests, structural failure occurred within the sealing zone; no cracks or slippage were observed in the clamping area.

**Figure 9:**
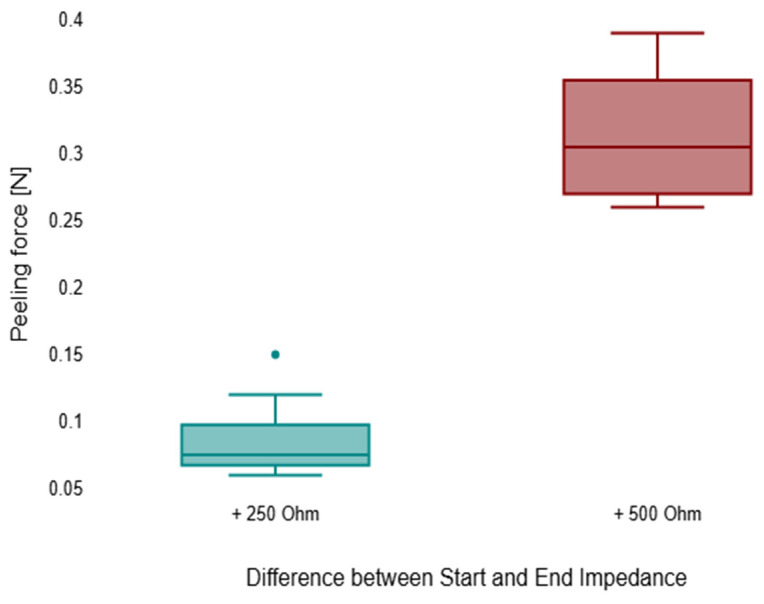
Box plot of the mean peeling forces as a function of the difference between initial and final impedance, a constant compression pressure of 2.5 kg, and 12 Aortic strip preparations each (own results).

On 12 aortic strip preparations at a compressive force of 2.5 kg, and difference between initial and final resistance of 250 Ω and 500 Ω, respectively, peel forces were determined. At a 250 Ω setting, they were 0.09 ± 0.03 N, and at a 500 Ω setting, they rose to 0.32 ± 0.05 N. This corresponds to an increase of + 25.5%. Table 3 and Figure 9 show an overview of these results. Finally, tests were carried out with an increased compression pressure of 5 kg and a difference between initial and final impedance settings of 500 Ω. An average peel force of 0.91 ± 0.2 N was determined. The minimum peel force was 0.09 N, and the maximum peel force was 1.36 N.

**Figure 10:**
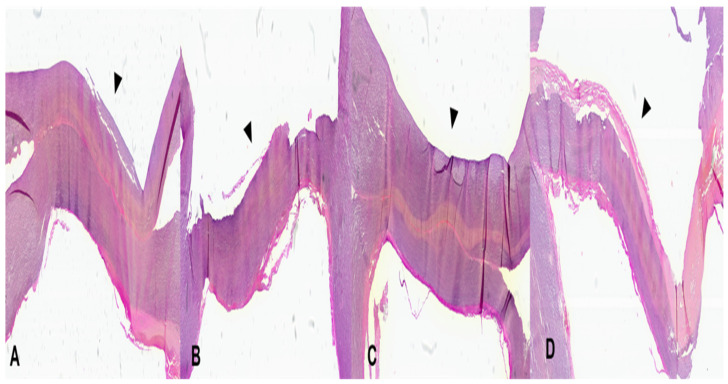
Histological representation of the sealing sutures (see arrow marking) depending on the compressive forces (**A**) 2.5 kg, (**B**) 5 kg, (**C**) 7.5, and (**D**) 10 kg at a 4× magnification (Institute of Pathology, Marburg).

Compared to the other two groups, there was a clearly significant difference (*p* < 0.001 ***). Figure 10 shows histological examinations of the sealing sutures at different compression forces. Despite bipolar current flow, the original wall structures of the aortic walls were preserved. Coagulated sealing zones were formed in the contact zone of the aortic strips. Histological evaluation confirmed preservation of the vessel wall architecture.

## 4. Discussion

This study provides the first systematic analysis of the sealability of elastin-rich aortic wall strips from pigs under varying mechanical and electrical parameters. The results show that bipolar sealing of the aortic wall is not successful under standard conditions. On the other hand, a significant improvement in peeling forces can be achieved under defined compression pressures and with adapted impedance control. This work thus contributes significantly to the understanding of the possibilities and limitations of energy-assisted sealing technologies in elastin-rich tissues.

With an elastin content of over 40%, aortic walls have a tissue structure that differs fundamentally from collagen-rich vessels. The elastin properties have a high elasticity, pronounced resilience, and remarkable thermal stability [18]. While collagen denatures even at moderate temperatures, thereby forming the basis for stable sealing sutures, elastin remains largely unchanged in structure even at higher temperatures. These properties make it considerably more difficult to form a homogeneous coagulation zone and may explain why aortic wall sealing was ineffective under standard conditions. The combination of high elasticity, low compressibility, and thermal resistance is perhaps crucial in this regard.

While carotid arteries can be almost completely compressed under high forces, the aortic wall under the same physical conditions achieves less than half of this compression. However, since mechanical compression is an important prerequisite for bipolar sealing, low compressibility is a significant limiting factor. The aorta, being an elastic artery, exhibits lower thermal compression and thus poorer sealing quality due to its high elastin content, high compliance, and heterogeneous wall structure. The carotid artery, as a muscular artery with a high proportion of smooth muscle cells (SMC) and collagen, can be compressed much more effectively, exhibits a clearly defined coagulation zone, and achieves higher mechanical stability (burst pressure and peel force).

Only defined compression using our specially developed device led to reproducible and significant improvements in sealing quality. With increasing compression pressure, the peel forces achieved increased significantly, indicating that mechanical compression not only has a supportive effect but is also a prerequisite for the successful sealing of elastin-rich tissue.

The observed effects can be explained by several mechanisms. Higher compression improves contact between the tissue layers, reduces the fluid content, and stabilizes the tissue position, making energy transfer more homogeneous. At the same time, denser tissue structure leads to lower heat dissipation and thus to higher local temperatures, which are necessary for collagen denaturation. These mechanisms may explain why sealing failed under standard conditions, while defined compression pressures led to reproducible and significant improvements.

The peel forces measured are within the range of mechanical strengths described in the literature for collagen-rich vessels, thereby confirming that tissues with a high collagen content form particularly stable thermal bonds [23,28]. In comparison, however, elastin-rich arteries consistently exhibit lower peel force values. This reflects their structural properties: Under thermal stress, elastin does not form load-bearing intermolecular bridges, whereas collagen fibers form robust bonds through denaturation and recombination. Particularly, the ratio of collagen to elastin seems to fundamentally limit maximum achievable mechanical bonding following bipolar sealing.

Furthermore, variation in the difference between the initial and final impedance settings significantly influenced sealing quality. The increase from 250 Ω to 500 Ω led to a significant increase in peel forces at constant compression pressure.

Presently, impedance control of modern sealing systems assumes that the electrical conductivity of the tissue decreases during coagulation. A higher resistance level setting between the beginning and end of the sealing procedure leads to energy being applied more intensively before the device automatically terminates the process. This is particularly important for elastin-rich tissue, as elastin requires a higher amount of energy to undergo structural changes due to its thermal stability. These results suggest that the standard impedance thresholds of many devices are insufficient for elastin-rich tissues and that parameter adjustment is necessary to create a stable coagulation zone. A combination of increased compression pressure and increased impedance delta resulted in the highest measured peel forces and shows that mechanical and electrical parameters in this case work synergistically.

Histological examinations of the seal sutures confirm the mechanical results. Despite energy input, the essential wall structures of the aortic wall were preserved, and coagulation zones were limited to the contact surfaces of the aortic strips. The elastic lamellae of the media remained largely intact, underscoring the thermal resistance of elastin. These findings explain why the sealing seams had only limited load-bearing capacity despite energy input. At the same time, they show that sealing occurs primarily through collagen denaturation and mechanical fixation effects, while elastin plays a minor role. Although the overall architecture remains intact, microstructural changes such as elastin-related fragmentation or weakening of the medial lamellae cannot be ruled out.

Like any experimental study, this study has limitations. The investigations were performed ex vivo, so that in vivo relevant factors such as perfusion, temperature, or wall tension could not be considered. Histological analysis was deliberately qualitative in order to assess structural integrity and localization of the coagulation zones. It showed that vascular architecture was largely preserved and that coagulation was limited to the contact surfaces. A quantitative morphometric analysis: thermal lesion depth, elastin fragmentation, or collagen-related remodeling was not performed as part of this study. It would, of course, further strengthen the correlation between microstructural changes and mechanical performance. Quantitative methods (e.g., polarization microscopy, multiphoton imaging, morphometric segmentation) are planned for future studies.

Furthermore, only selected parameters were examined, while other influencing factors, such as electrode geometry, energy profiles, or contact times, could perhaps provide additional insights. As an ex vivo strip model was employed, in vivo-relevant factors such as perfusion, temperature gradients, wall tension, or hemodynamic stress could not be taken into account. The results should therefore be interpreted as an exploratory mechanistic analysis and not as a functional assessment of clinical sealing performance. Peel-force testing provides a reproducible measure of tissue adhesion but does not directly reflect clinically relevant sealing performance. Functional factors were not included in this ex vivo model. The applicability of our results to surgical use is therefore limited. Additional studies on intact, pressurized vessels and hemostatic function analyses are required. Although peel force is established as the sole parameter for evaluating seal quality, it could be supplemented by further mechanical tests, such as burst pressure tests or tensile tests. In addition, tests on intact vessel segments are planned. The peel-force values obtained in this study cannot be equated with the performance of established commercial vessel-sealing systems. Instead, they highlight mechanistic trends in elastin-rich tissue, where the inherently low collagen content supposedly limits maximum achievable bonding. The results, therefore, provide structural and biophysical insight rather than clinical performance thresholds.

Our mechanistic explanations—in particular, the role of low tissue compressibility, high thermal stability of elastin, and the significance of an expanded impedance interval—are meant as hypothesis-generating interpretations. Direct measurements of temperature distributions, electrical conductivity, collagen-related denaturation processes, or structural changes in elastic lamellae were not performed. Accordingly, our mechanistic conclusions remain inference-based. Future work will integrate quantitative thermal and structural analyses to experimentally substantiate the hypotheses formulated here.

Overall, the results show that resilient bipolar sealing of elastin-rich aortic walls is fundamentally possible, but only under specific conditions. Mechanical compression and impedance control are crucial parameters that must be adapted for elastin-rich tissue. The study thus provides an important basis for further concepts regarding energy-assisted sealing technologies and may, in the long term, contribute to improving the safety and effectiveness of surgical procedures involving large vessels. To further expand on our findings, several complementary analytical methods are available, which could more precisely characterize both thermal tissue damage and microstructural integrity of sealed vessels. Systematic quantification of thermal injury depth would allow for objective assessment of coagulation-induced tissue changes as a function of temperature, compression, and vessel type. In addition, polarization microscopy could be used to visualize collagen fiber architecture and its thermally induced denaturation. This would provide valuable structural information, particularly for comparing elastic and muscular arteries. Furthermore, multiphoton imaging (SHG/2PEF) offers the possibility of non-invasively assessing the ultrastructural integrity of collagen and elastin. Additional staining would not be necessary, and fusion quality could be quantified at the submicroscopic level. Together, these methods could provide a significantly more comprehensive picture of the thermal and structural changes following bipolar vessel sealing. Mechanical outcomes (burst pressure, peel force) could be substantiated mechanistically.

Nevertheless, the study presented provides an experimental model that will enable future investigations into the optimization of sealing parameters, electrode geometries, and energy profiles.

## 5. Conclusions

This study makes an important mechanistic contribution to tissue-specific sealing in surgical practice. Results show that the effectiveness of bipolar vessel sealing may depend significantly on the structural composition of vessel walls. Arteries rich in elastin present lower mechanical stability, and their low thermal bonding capacity may have a significant role in this. Collagen-rich vessel sealings give the impression of being significantly more robust. Though probably important, collagen as well as elastin content may not be unique factors. Nevertheless, these findings underscore the need to better adapt sealing technologies to specific vascular architecture. As this study was limited to ex vivo peel-force testing, results should be viewed as mechanistic rather than functionally validated. Additional biomechanical and histological analyses will be required before clinical conclusions can be drawn with certainty. Energy- and/or pressure-adaptive systems that deliver reliable results even in both collagen-rich and elastic arteries would advance procedures.

## Figures and Tables

**Table 1:** Average percentage compression of aortic walls (*n* = 12) and carotid arteries < 5 mm (*n* = 12) as a function of compression pressure [N].

Compression Pressure [N]	0	9.5	27.3	73.5	98	196	245	294	343	392	441	490
**Mean compression % of the aortic wall**	0	6.2 ± 0.03	14.6 ± 0.04	18.5 ± 0.04	21.3 ± 0.04	27.5 ± 0.04	30.3 ± 0.05	33.1 ± 0.05	36.5 ± 0.05	38.7 ± 0.04	42.1 ± 0.05	44.9 ± 0.05
**Mean compression % of the** **carotid artery < 5 mm**	0	7.1 ± 2.7	20.0 ± 2.7	28.6 ± 3.1	35.7 ± 3.2	51.4 ± 3.3	58.6 ± 2.9	65.7 ± 3.2	72.6 ± 3.8	78.6 ± 3.4	87.4 ± 4.1	92.8 ± 3.2

**Table 2:** Overview of the average peeling forces of aortic strips (*n* = 12 in each case) at different compression forces.

Compression Force [kg]	2.5	5.0	7.5	10
Mean peeling force [N]	0.09	0.19	0.52	0.71
±SE	0.03	0.07	0.11	0.18
Min [N]	0.06	0.13	0.27	0.43
Max [N]	0.15	0.34	0.65	0.95

**Table 3:** Comparison of the average peeling forces for two different impedance deltas [Δ], examined on 12 aortic strip preparations each.

Δ–Impedance [Ω]	250	500
Peeling force [N]	0.09	0.32
±SD	0.03	0.05
Min [N]	0.06	0.26
Max [N]	0.15	0.39

## Data Availability

The data presented in this study are openly available in [Kirschbaum A.] [https://doi.org/10.5281/zenodo.19640379] [https://doi.org/10.5281/zenodo.19640378].
